# Simple analytical model of the effect of high pressure on the critical temperature and other thermodynamic properties of superconductors

**DOI:** 10.1038/s41598-018-26029-9

**Published:** 2018-05-16

**Authors:** Mateusz Krzyzosiak, Ryszard Gonczarek, Adam Gonczarek, Lucjan Jacak

**Affiliations:** 1 University of Michigan–Shanghai Jiao Tong University Joint Institute, 800 Dongchuan Rd, Shanghai, 200240 China; 2 Faculty of Fundamental Problems of Technology, Wrocław University of Technology, Wybrzeże Wyspiańskiego 27, 50-370 Wrocław, Poland; 3 Faculty of Computer Science and Management, Wrocław University of Technology, Wybrzeże Wyspiańskiego 27, 50-370 Wrocław, Poland

## Abstract

Within the general conformal transformation method a simplified analytical model is proposed to study the effect of external hydrostatic pressure on low- and high-temperature superconducting systems. A single fluctuation in the density of states, placed away from the Fermi level, as well as external pressure are included in the model to derive equations for the superconducting gap, free energy difference, and specific heat difference. The zero- and sub-critical temperature limits are discussed by the method of successive approximations. The critical temperature is found as a function of high external pressure. It is shown that there are four universal types of the response of the system, in terms of dependence of the critical temperature on increasing external pressure. Some effects, which should be possible to be observed experimentally in *s*-wave superconductors, the cuprates (*i*.*e*. high-*T*_c_ superconductors) and other superconducting materials of the new generation such as two-gap superconductors, are revealed and discussed. An equation for the ratio ${{\boldsymbol{ {\mathcal R} }}}_{{\bf{1}}}$ ≡ 2Δ(0)/*T*_c_, as a function of the introduced parameters, is derived and solved numerically. Analysis of other thermodynamic quantities and the characteristic ratio ${{\boldsymbol{ {\mathcal R} }}}_{{\bf{2}}}$ ≡ Δ*C*(*T*_c_)/*C*_N_(*T*_c_) is performed numerically, and mutual relations between the discussed quantities are investigated. The simple analytical model presented in the paper may turn out to be helpful in searching for novel superconducting components with higher critical temperatures induced by pressure effects.

## Introduction

A revival of theoretical studies on a new generation of superconducting materials has been recently brought by the discovery of iron-based superconductors^1,2^. Efforts on the theoretical front line have been accompanied, and in many cases driven, by increasingly better characterization techniques. Recent studies on novel superconducting materials have also focused on high-*T*_c_ copper-oxide quasi two-dimensional superconducting systems that are particularly promising in application-based solutions. Other materials of particular interest include various doped superconducting compounds such as spinel- and perovskite-type structures of superconducting compounds of a trivalent rare-earth and a divalent alkali-earth ion. Superconducting compounds of MgB_2_ with a C, Al or Sc substitution, or organic superconductors with controlled bandwidth and band filling have also been actively studied in recent years^3–12^. In particular, theoretical research, supported by experimental data, points to the fact that applying high external pressure after the dissociation process in H_3_S, results in the onset of a superconducting state with the transition temperature of 203 K^13–19^.

An important tool for quantitative theoretical studies of superconducting systems is the gap equation, accompanied by the carrier concentration equation, and analysis of the free energy^3,4,20–26^. The gap equation appears in similar forms in the BCS-theory, the Eliashberg formalism, and the Van Hove scenario, with the latter taking into account the low-dimensional structure of high-*T*_c_ materials, implying the presence of fluctuations in the density of states. Theoretical description of such superconducting systems usually requires to take into account spin-fluctuations or strong-correlation effects. These can be included by means of an effective Hamiltonian of the strongly-interacting Hubbard model, with a given (multiband) one-particle dispersion relation enriched by self-energy corrections, fitted carriers concentration, and a quite general form of the pairing potential. The latter can be decomposed into an antisymmetric and a symmetric part determining the symmetry of the order parameter^27–38^.

In order to address some of the above problems in a systematic analytical manner, the framework of the conformal transformation method can be used, especially if the behavior of a superconducting system at the zero- or sub-critical temperatures is of particular interest^5–8,39–41^. This approach allows one to derive some original fundamental relations for basic parameters characterizing a superconducting system, such as the energy gap, critical temperature, free energy difference, and heat capacity jump as functions of a single fluctuation in the density of states. This fluctuation (a peak) in the density of states is located a certain distance *x*_0_ from the Fermi level. With an additional parameter *χ*, characterizing the height of the fluctuation, the effect of an external pressure *p* can be incorporated in the model by means of the set of three parameters *χ*, *x*_0_, and *p*. For a comprehensive list of symbols used in the paper, please refer to Table I in the Appendix.

Within the proposed model, it is also possible to study the relation between the energy gap Δ at *T* = 0 and the transition temperature *T*_c_, quantified by the ratio ${ {\mathcal R} }_{1}\equiv 2{\rm{\Delta }}(0)/{T}_{{\rm{c}}}$. Another characteristic ratio ${ {\mathcal R} }_{2}\equiv {\rm{\Delta }}C({T}_{{\rm{c}}})/{C}_{{\rm{N}}}({T}_{{\rm{c}}})$, where Δ*C*(*T*_c_) = *C*_S_(*T*_c_) − *C*_N_(*T*_c_), defines the leap of the heat capacity between the superconducting and the normal phase at the transition temperature. The fundamental thermodynamic quantities, and hence the ratios ${ {\mathcal R} }_{1}$ and ${ {\mathcal R} }_{2}$, are measured in experimental studies of superconductors and can be easily compared against theoretical results.

In the approach presented in this paper we do not consider the cases when a *d*-wave superconducting state is realized in the system, or when phase transitions with a discontinuous order parameter, or regions of the pseudogap, emerge in the system^4,8,41,42^.

## Formalism

In order to discuss the effect of high pressure on a superconducting system we use the conformal transformation method^40^, that we have developed in our previous papers^3,6–8,27,43–45^.

The conformal transformation is a mathematical procedure which can be applied to an arbitrarily complex model of a *s*-wave and *d*-wave superconductor, high-*T*_c_ cuprates, or more complicated systems such as two-gap superconductors, set within the framework of mean-field approximation with a certain pairing potential. The method transfers the model from the original reciprocal (momentum) space to an isotropic space, where all properties of the system included in the dispersion relation are transferred to the scalar field of the density of states. Consequently, the pairing potential, which in general has a spin-antisymmetric or a spin-symmetric structure, becomes expressed in terms of a double series of spherical or Fourier harmonics indexed by the number *l* for 3D or 2D systems, respectively. For the spin-antisymmetric part, only the harmonics with even values of *l* are included, whereas for the spin-symmetric part – only those with odd values.

In most of various approaches used to explain different properties of superconductors of the new generation, including those with high critical temperatures^46^, a single microscopic mechanism responsible for high-*T*_c_ superconductivity is assumed. However, such mechanism has not been equivocally identified yet. Some of recent ideas include a mechanism based on coupling through electron-electron interaction^47^, or spin exchange^48,49^, as well as a modified phonon-mediated mechanism^50,51^ proposed by Anderson^52,53^ and based on the concept of the resonating valence bond states. On the other hand, in all of these approaches it is commonly assumed that whatever the mechanism is, it results in formation of Cooper pairs.

The effective interaction between fermion charge carriers in strongly correlated systems is quite complicated, as it in general depends on both the spin of charge carriers and the current they carry. Because of the expected magnetically-mediated nature of pairing, arising from exchange of magnetic fluctuations, the conventional phonon-mediated pairing mechanism raises doubts. Therefore, we will assume that a generic, boson- mediated, strongly anisotropic attraction mechanism provides the required pairing potential.

The advantage of the conformal transformation method is that some effects, such as, for example, particle-hole asymmetry and the spectral function defined in the Eliashberg equations can be also included. The final product of the conformal transformation method is then a set of equations which possess the symmetry of the original reciprocal space. Considering the symmetry group of that space, which usually is a non-abelian group, one can separate its elements into several equivalence classes and find the corresponding irreducible representations. Consistently, there must always exist basis functions gathered in subsets, containing one or a few functions, being invariants of the symmetry group. Hence, for a fixed harmonic *l* (of the spherical or the Fourier double-series expansion), the allowed structure of the energy gap must be defined be a combination of the basis functions of invariant subsets. Nevertheless, there always exists a trivial irreducible representation corresponding to the one-element invariant subset representing the identity element. This subset determines the structure of the *s*-wave energy gap, for which *l* = 0 and the suitable spherical and Fourier harmonic is just a constant function.

The final form of the set of equations, after the conformal transformation is applied, reveals the symmetry group of the original reciprocal space, and includes a quite complicated dimensionless scalar field of the density of states. These equations, in general, are related by the common form of the energy gap. However, in the limit *T* → *T*_c_, the energy gap Δ = 0, and all equations of this set become separated.

In the present discussion, our starting point is a set of two equations for a pressure-free system (*p* = 0), derived within the Green function formalism in the mean-field approximation. One of these equations is the momentum-space gap equation

1 $${{\boldsymbol{\Delta }}}_{{\bf{k}}}=\sum _{{\bf{k}}{\boldsymbol{^{\prime} }}}\,V({\bf{k}},{\bf{k}}^{\prime} )\frac{{{\boldsymbol{\Delta }}}_{{\bf{k}}^{\prime} }}{{E}_{{\bf{k}}^{\prime} }}\,\tanh \,\frac{{E}_{{\bf{k}}^{\prime} }}{2T},$$

where ${E}_{{\bf{k}}}=\sqrt{{({\xi }_{{\bf{k}}}-\mu )}^{2}+{{\boldsymbol{\Delta }}}_{{\bf{k}}}^{2}}$. It is supplemented by another self-consistent equation for carrier concentration

2 $$n=\frac{1}{N}\,\sum _{{\bf{k}}}\,(1-\frac{{\xi }_{{\bf{k}}}-\mu }{2{E}_{{\bf{k}}}}\,\tanh \,\frac{{E}_{{\bf{k}}}}{2T}),$$

where *N* denotes the number of lattice sites. Eq. (2) determines the chemical potential *μ*, and allows us to express *μ* in Eq. (1) in dependence on the conduction band filling *n* (defined for the normal phase at *T* = 0). The transformed dispersion relation appearing in Eqs (1) and (2) is usually defined with respect to the Fermi level. Therefore, within this approach it is also possible to study superconducting systems with a partially-filled conduction band. It can also be applied to anisotropic superconducting systems with an arbitrary dispersion relation for spin-singlet *s*-wave, and *d*-wave symmetry states, as well as for the spin-triplet *p*-wave symmetry state, including the high-*T*_c_ superconducting cuprates, with a given symmetry defined in the reciprocal space (or transformed to that space). Other factors, such as the carrier concentration, and the pairing potential amplitudes in the singlet and the triplet paring channels *V*_0_ and *V*_1_ can also be discussed. It is possible to study the stability of these symmetry states in more complicated scenarios^3,8,35,41,43,44,54,55^. Here, however, we formulate a simple model, which appears as a result of reduction of more involved models of novel low- or high-*T*_c_ superconductors. In the approach outlined in the present paper, we confine the discussion to the case which can be considered as equivalent to a class of spin-singlet *s*-wave superconducting systems, characterized by one constant pairing potential amplitude *g* defined as below.

### Gap equation and free energy

Let us consider an anisotropic superconducting system with a fixed carrier concentration. We assume a generic form of the dispersion relation *ξ*_**k**_, and pressure *p* = 0, and apply the conformal transformation method developed in refs^3,27,44^. Next, restricting the pairing potential to exactly one harmonic *l* = 0, with amplitudes *V*_0_ = *g* and *V*_1_ = 0 being consistently fixed as well, a spin-singlet *s*-wave is formed, and the gap equation (1) reduces to the form

3 $$\frac{1}{g}=\frac{{\nu }_{0}}{2}\langle {\int }_{-{\xi }_{{\rm{p}}}}^{{\xi }_{{\rm{p}}}}\,\frac{d\xi \,{\mathscr{K}}(\xi ,\omega )}{2\sqrt{{\xi }^{2}+{{\rm{\Delta }}}^{2}}}\,\tanh \,\frac{\sqrt{{\xi }^{2}+{{\rm{\Delta }}}^{2}}}{2T}\rangle ,$$

where *ν*_0_ is the density of states of the BCS type. The so-called cut-off parameter *ξ*_p_ is determined individually for each superconducting system and depends on the pairing mechanism as discussed above^40^. It corresponds to the Debye energy (*k*_B_*T*_D_) for systems with electron-phonon pairing potential. The symbol 〈…〉 denotes averaging over planar or spherical angles *ω* in the two- or three-dimensional space, respectively^27,40^. Since the dimensionless scalar field of the density of states ${\mathscr{K}}(\xi ,\omega )$ is the only function depending on *ω* in the equation, after averaging, Eq. (3) simplifies to

4 $$\frac{1}{g}=\frac{{\nu }_{0}}{2}\,{\int }_{-{\xi }_{{\rm{p}}}}^{{\xi }_{{\rm{p}}}}\,\frac{d\xi \,{\mathscr{N}}(\xi )}{2\sqrt{{\xi }^{2}+{{\rm{\Delta }}}^{2}}}\,\tanh \,\frac{\sqrt{{\xi }^{2}+{{\rm{\Delta }}}^{2}}}{2T},$$

where ${\mathscr{N}}(\xi )=\langle {\mathscr{K}}(\xi ,\omega )\rangle$ is the dimensionless density of states, being the final product of the conformal transformation approach. Examples of analytical formulas for the density of states in the 2D tight-binding model, with the nearest-neighbor and the next-nearest-neighbor integrals taken into account, are given in ref.^27^. Note that effects of the particle-hole asymmetry and some extra factors, such as *e*.*g*. the spectral function defined in Eliashberg equations, can be directly included in ${\mathscr{N}}(\xi )$ as well. Because in the BCS-like model ${\mathscr{N}}(\xi )=1$, we assume it in the form ${\mathscr{N}}(\xi )=1+\rho (\xi )$. According to previous studies^45^, the function *ρ* can substantially change various parameters of a superconducting system, especially if the function has a narrow fluctuation (a peak) in the vicinity of the Fermi level. Moreover, the results obtained for peaks of various shapes yield similar results, with differences of the order of a few per cent^39^. Therefore, at this point, we do not need to consider any particular analytical properties of the function *ρ*, treating it as a locally constant function.

In the next step, introducing the symbols: *x* = *ξ*/2*T*, *x*_p_ = *ξ*_p_/2*T*, and *D* = Δ/2*T*, we rewrite Eq. (4) as

5 $$\frac{1}{g}=\frac{{\nu }_{0}}{2}\,{\int }_{-{x}_{{\rm{p}}}}^{{x}_{{\rm{p}}}}\,\frac{dx\,[1+\varrho (x)]}{\sqrt{{x}^{2}+{D}^{2}}}\,\tanh \,(\sqrt{{x}^{2}+{D}^{2}}),$$

where the function *ρ*(*ξ*) has been redefined into $\varrho (x)$, which can be again modelled in various ways. The factor *g* can be eliminated by taking into account Eq. (5) in the limit cases *T* = *T*_c_ and *D* = 0.

Employing results given in refs^20,35^, one can easily reproduce the free energy difference Δ*F* = *F*_S_ − *F*_N_ between the superconducting and the normal phase as

6 $${\rm{\Delta }}F={\int }_{0}^{\Delta }\,d{\rm{\Delta }}^{\prime} \{\frac{{\rm{\Delta }}^{\prime} }{g}-\frac{{\nu }_{0}}{2}\,\,{\int }_{-{x}_{{\rm{p}}}}^{{x}_{{\rm{p}}}}\,dx\frac{[1+\varrho (x)]\,{\rm{\Delta }}^{\prime} }{\sqrt{{x}^{2}+{({\rm{\Delta }}^{\prime} /2T)}^{2}}}\,\tanh \,(\sqrt{{x}^{2}+{({\rm{\Delta }}^{\prime} /2T)}^{2}})\}.$$

Hereafter, we take into account a model form of $\varrho (x)$, which so far has been treated as constant in local intervals. Now, the function $\varrho (x)$ should have a narrow fluctuation (a peak) which is shifted a distance *x*_0_ from the Fermi level. Since various particular forms of the fluctuation produce similar results, we propose to take the function $\varrho (x)$ in the following form

7 $${\varrho }_{j}(x)=\chi \frac{(2j-1)!!}{{2}^{j-1}\,(j-1)!}\,{\cosh }^{-2j}\,(x-{x}_{0})\,,$$

where *x*_0_ is the point, away from the Fermi level, where ${\varrho }_{j}(x)$ is maximum. This point can be either below the Fermi level (for *x*_0_ < 0) or above it (for *x*_0_ > 0). The effect of such a fluctuation on the enhancement of the critical temperature is strongly suppressed^45^.

The parameter *χ* fixes the height of the local fluctuation, which can be positive as well as negative when the averaged fluctuations away from the point *x*_0_ are higher than those at the very point *x*_0_. The half-width of the fluctuation is denoted as $\eta =2\,{\rm{arccosh}}\,(\sqrt[2j]{2}\,)$.

Moreover, for *j* → ∞ the function ${\varrho }_{j}(x)\to 2\chi \delta (x-{x}_{0})$, where *δ*(*x*) is the Dirac delta^56,57^. The case *χ* = 0 corresponds to the BCS-like model, *i*.*e*. the free-electron model with restricted pairing interaction near the Fermi level. The pairing interaction is characterized by two independent parameters *g* and *x*_p_, which should be taken with reference to real superconducting materials. Then one can compare thermodynamic quantities in the BCS-like model with those in the approach under discussion.

## External Hydrostatic Pressure

The superconducting state is formed in a fermion-carrier system that is located in the interior of a superconducting sample. The structure of the superconducting material determines many properties of the carrier system through the (usually complicated) form of their dispersion relation. External pressure imposed on the superconducting sample, causes the sample, as well as the system of fermion carriers, to stabilize under new conditions. This new equilibrium, in particular, defines a modified dispersion relation of carriers, parametrized now by the external pressure *p*. In more detail, applying high pressure *p* to a superconducting sample, supplies an extra energy to each unit cell, which may consist of one or a few atoms. The amount of that additional energy is *vp*, where *v* is the specific volume, defined in the real space, occupied by a charge carrier. The supplied energy is absorbed by unit cell atoms, and hence a new equilibrium state is reached with the dispersion relation *ξ*_**k**_(*p*), parametrized also by *p*. Therefore, we can treat *ξ*_**k**_(*p*) as a function of the wave vector **k** and the pressure *p* and, expand it in a series

8 $${\xi }_{{\bf{k}}}(p)={\xi }_{{\bf{k}}}(0)+Q\,p\,,$$

where

$$Q={[\frac{\partial }{\partial p}{\xi }_{{\bf{k}}}(p)]}_{{\bf{k}}={{\bf{k}}}_{{\rm{F}}},p=0},$$

and *ξ*_**k**_(0) is the dispersion relation for the pressure-free system. Since applying high pressure to a superconducting material can merely cause a small linear increase of the energy of unit cell atoms by *vp*, it will induce a proportional linear increase of the charge carrier energy as well. Therefore, we state that *Qp* > 0 should be proportional to, or rather should be of the order of, $$({m}^{\\bigstar }/{M}_{{\rm{A}}})\,vp$$, where $${m}^{\\bigstar }$$ is the effective mass of the charge carrier and *M*_A_ is the total mass of unit cell atoms. Hence, for example, assuming *p* ~ 10^9^ Pa, *v* ~ 10^−28^ m^3^, $${m}^{\\bigstar }/{M}_{{\rm{A}}}\sim {10}^{-5}$$ and *k*_B_ = 1, 381 10^−23^ J/K, we get a rough estimate of *Qp* ~ 0.1 K in the temperature scale. Therefore, $Q\,p\ll {x}_{{\rm{p}}}$, since *x*_p_ is identified with the Debye temperature for superconductors with electron-phonon pairing potential.

Consequently, we can follow the conformal transformation method and repeat all its steps with the dispersion relation *ξ* = *ξ*_**k**_(*p*) − *Qp*. Eventually, Eq. (5) for *p* > 0 assumes the form

9 $$\frac{1}{g}=\frac{{\nu }_{0}}{2}\,{\int }_{-{x}_{{\rm{p}}}}^{{x}_{{\rm{p}}}}\,\frac{dx\,[1+{\varrho }_{j}(x)]}{\sqrt{{(x+\kappa p)}^{2}+{D}^{2}}}\,\tanh \,[\sqrt{{(x+\kappa p)}^{2}+{D}^{2}}],$$

where *κ* = *Q*/2*T*. Although the local fluctuation is precisely mapped by ${\varrho }_{j}(x)$ for fixed *j* and *χ*, in ref.^39^ we show that for *j* > 100, corresponding to narrow fluctuations, numerical and analytical results coincide with the case *j* → ∞, and can be better fitted by the self-correcting parameter *χ* within the applied model. Hence, furthermore, we replace ${\varrho }_{j}(x)$ by 2*χδ*(*x* − *x*_0_).

### Critical temperature under high pressure

For a system at the critical temperature *T* = *T*_c_, when Δ = 0, gap equations derived *e*.*g*. for *s*- and *d*-wave superconductors, become separated from each other, and can be considered independently. Note that for all cases other than pure *s*-wave, the scalar field of density of states is averaged with the spherical harmonics or the Fourier harmonics, yielding effectively also a density-of-states-type function, that can be modelled similarly to a standard density of states function.

Then, including a single fluctuation in the form 2*χδ*(*x* − *x*_0_) in Eq. (9) and neglecting terms of the order of (*Qp*/*ξ*_p_)^2^, we find the critical temperature as a function of *χ* and *p*, for a given value of *x*_0_, in the form

10 $${T}_{{\rm{c}}}(\chi ,{x}_{0},p)={T}_{{\rm{c}}}(0,0,0)\,\exp \,\{\chi \,\frac{\tanh \,[\tau ({x}_{0}+\kappa p)]}{\tau ({x}_{0}+\kappa p)}\}\,.$$

In order to make sure that the values of *x*_0_ and *κ* are fixed in calculations, it is assumed that they are defined with respect to the critical temperature for a pressure-free system (*p* = 0), which is denoted as *T*_c_(*χ*, *x*_0_, 0). Hence

$$\tau \equiv \tau (\chi ,{x}_{0},p)=\frac{{T}_{{\rm{c}}}(\chi ,{x}_{0},0)}{{T}_{{\rm{c}}}(\chi ,{x}_{0},p)}\,$$

is a function of *p*, and *τ* = 1 for *p* = 0. As a result of this step, Eq. (10) becomes implicit, with the function *T*_c_(*χ*, *x*_0_, *p*) on both sides of the equation. However, it can be transformed to a more convenient form

11 $${T}_{{\rm{c}}}(\chi ,{x}_{0},p)={T}_{{\rm{c}}}(\chi ,{x}_{0},0)\,\exp \,\{\chi \,[\frac{\tanh \,[\tau ({x}_{0}+\kappa p)]}{\tau ({x}_{0}+\kappa p)}-\frac{\tanh \,{x}_{0}}{{x}_{0}}]\}.$$

The function $\tanh (x)/x$ is even and positive for all real *x*, and decreases from 1 to 0 as |*x*| varies from 0 to ∞. Therefore, for *x*_0_ < 0 the critical temperature *T*_c_(*χ*, *x*_0_, *p*) achieves a maximum for a fixed *χ* > 0 and a minimum for *χ* < 0, if *p* = −*x*_0_/*κ*. Hence, with respect to the case *p* = 0, we have

12 $${T}_{{\rm{c}}}(\chi ,{x}_{0},-{x}_{0}/\kappa )={T}_{{\rm{c}}}(\chi ,{x}_{0},0)\,\exp \,[\chi \,(1-\frac{\tanh \,{x}_{0}}{{x}_{0}})].$$

**Figure 1:**
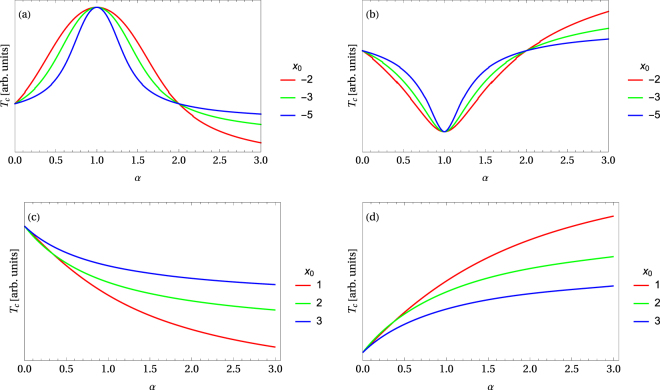
Four possible types of the dependence of the critical temperature on high external pressure, where *α* = *κp*/|*x*_0_| is a dimensionless positive scaling parameter for pressure: (**a**) *T*_c_(1) > *T*_c_(0) and *x*_0_ < 0, then *χ* > 0, (**b**) *T*_c_(1) < *T*_c_(0) and *x*_0_ < 0, then *χ* < 0, (**c**) only *T*_c_(0) is given, *x*_0_ > 0 and *χ* > 0, (**d**) only *T*_c_(0) is given, *x*_0_ > 0 and *χ* < 0. It is shown that the shape of the curves changes, depending on the parameter *x*_0_.

On the other hand, if *x*_0_ ≥ 0, according to Eq. (11), the applied pressure drives the critical temperature downwards *T*_c_(*χ*, *x*_0_, *p*) ≤ *T*_c_(*χ*, *x*_0_, 0) if *χ* > 0 or upwards *T*_c_(*χ*, *x*_0_, *p*) ≥ *T*_c_(*χ*, *x*_0_, 0) if *χ* < 0. Consequently, for the four possible combinations of parameters *x*_0_ < 0, *x*_0_ > 0 and *χ* > 0, *χ* < 0, four types of response to the applied pressure are possible, as far as the critical temperature is concerned. These four cases are illustrated in Fig. 1.

**Figure 2:**
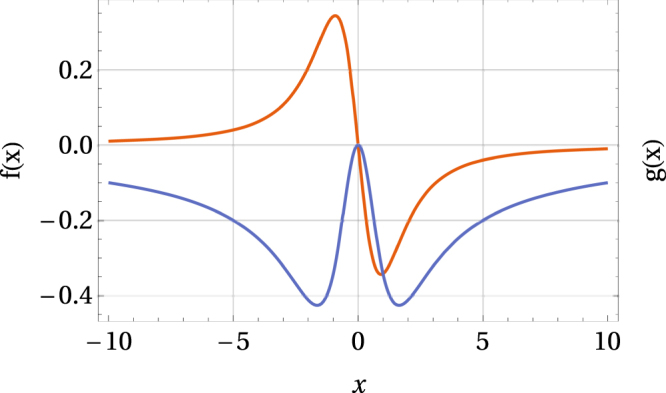
Functions *f* (red curve) and *g* (blue curve).

Moreover, from Eq. (11) we can also derive the pressure coefficient (at zero pressure)

13 $${c}_{p}={\frac{d{T}_{{\rm{c}}}(\chi ,{x}_{0},p)}{dp}|}_{p=0}=\frac{\chi \kappa \,{T}_{{\rm{c}}}(\chi ,{x}_{0},0)\,f({x}_{0})}{1+\chi \,g({x}_{0})},$$

where

$$f(x)=\frac{x\,{\cosh }^{-2}\,x-\,\tanh \,x}{{x}^{2}},\,g(x)=\frac{x\,{\cosh }^{-2}\,x-\,\tanh \,x}{x},$$

and *f*(*x*) ≤ 0 if *x* ≥ 0 and *f*(*x*) ≥ 0 if *x* ≤ 0, and *f*(0) = 0, whereas *g*(*x*) ≤ 0 and *g*(0) = 0 as illustrated in Fig. 2.

Hence, *c*_*p*_ < 0 if *x*_0_ < 0 and *χ* < 0, *i*.*e*. for the type-(b) behavior, and *c*_*p*_ > 0 if *x*_0_ > 0 and *χ* < 0, *i*.*e*. for the type-(d) behavior, which is correct since the critical temperature is then a decreasing or an increasing function of pressure, respectively. However, for the type-(a) and type-(c) behavior, the sign of *c*_*p*_ may change although the critical temperature is an increasing or a decreasing function of pressure, respectively. Consequently, an additional condition 1 + *χg*(*x*_0_) > 0 must be imposed on the parameters *χ* and *x*_0_. Therefore, any detailed analysis requires the values of *χ*, *x*_0_ and *κ* to be known.

In order to fit the parameters introduced in the model, it is necessary to have at hand some experimental data for the critical temperature at pressure. Assuming that the critical temperatures under normal conditions *T*_c_(0), and the maximum or the minimum critical temperature *T*_c_(1) achieved under a high pressure *p*_*m*_ are known, based on Eq. (11), we can find the critical temperature for an arbitrary value of the pressure *αp* as

14 $${T}_{{\rm{c}}}(\alpha ,{x}_{0})={T}_{{\rm{c}}}(1){[\frac{{T}_{{\rm{c}}}(0)}{{T}_{{\rm{c}}}(1)}]}^{\frac{1-\frac{\tanh [\tau (1-\alpha ){x}_{0}]}{\tau (1-\alpha ){x}_{0}}}{1-\frac{\tanh {x}_{0}}{{x}_{0}}}},$$

where *α* ≥ 0, and *x*_0_ < 0 must be postulated. Here *T*_c_(0, *x*_0_) = *T*_c_(0) and *T*_c_(1, *x*_0_) = *T*_c_(1) are independent of *x*_0_. Moreover, *κ* = −*x*_0_/*p*_*m*_, and the critical temperature as a function of pressure *T*_c_(*p*) must be derived from the equation

$$t=\exp \,\{\mathrm{ln}\,[\frac{{T}_{c}(0)}{{T}_{c}(1)}]\cdot [\frac{1-\frac{t\,\tanh \,[{t}^{-1}(1-\alpha ){x}_{0}]}{(1-\alpha ){x}_{0}}}{1-\frac{\tanh \,{x}_{0}}{{x}_{0}}}-1]\},$$

where

$$p=-\,\frac{{x}_{0}}{\kappa }\alpha ={p}_{m}\alpha ,\,t=\frac{{T}_{{\rm{c}}}(p)}{{T}_{{\rm{c}}}(0)}.$$

Although the parameter *χ* is eliminated from Eq. (14), it can be derived back from Eq. (12), and the conditions *T*_c_(0) < *T*_c_(1) and *T*_c_(0) > *T*_c_(1) correspond to *χ* > 0 and *χ* < 0, respectively.

In the case *x*_0_ < 0, when the temperatures *T*_c_(0) and *T*_c_(1) at a high pressure *p*_*m*_ are known, the pressure coefficient *c*_*p*_ at *p* = 0 can be derived as a function of a single parameter *x*_0_ as

15 $${c}_{p}({x}_{0})=\frac{{T}_{{\rm{c}}}(0)}{{p}_{m}}\,\mathrm{ln}\,[\frac{{T}_{{\rm{c}}}(1)}{{T}_{{\rm{c}}}(0)}]\,h({x}_{0})\,{\{1-\mathrm{ln}[\frac{{T}_{{\rm{c}}}(1)}{{T}_{{\rm{c}}}(0)}]h({x}_{0})\}}^{-1},$$

where

$$h(x)=\frac{x\,{\tanh }^{2}\,x}{x-\,\tanh \,x}-1.$$

Note that *h*(*x*) = *h*(−*x*), *h*(*x*) > 0, *h*(0) = 2 and *h*(*x*) ~ |*x*|^−1^ when |*x*| → ∞.

Moreover, *c*_*p*_(*x*_0_) must be negative if *T*_c_(0) > *T*_c_(1), positive if *T*_c_(0) < *T*_c_(1), and it vanishes for large values of |*x*_0_|. When the temperatures *T*_c_(0) and *T*_c_(1) are given, and *T*_c_(0) > *T*_c_(1), the pressure coefficient *c*_*p*_(*x*_0_) is always negative, whereas *T*_c_(0) < *T*_c_(1), the pressure coefficient *c*_*p*_(*x*_0_) starts to be positive for values of *x*_0_ of sufficiently large magnitude. Therefore, small values of |*x*_0_| must be excluded.

Let us consider a superconducting system with a single fluctuation of the density of states shifted a distance *x*_0_ away from the Fermi level (where $|{x}_{0}|\ll {x}_{{\rm{p}}}$), where we also include the effect of high external pressure. As we have argued before, the external pressure contributes an extra (linear) term to the dispersion relation of charge carriers, with no changes to the density of states involved. For the pressure *p*_*m*_ ~ 10^9^ Pa, this extra term is of order 0.1 K. In terms of dimensionless quantities that we use in calculations, we normalize it by *T*_c_, *i*.*e*. the critical temperature for the system under discussion. Hence, *κp*_*m*_ ~ 0.1 and for the pressure of magnitude not exceeding 10^6^ Pa, no effect is expected. On the other hand, if $|{x}_{0}|\ll 1$, when the external pressure is not included, and since $\tanh (x)/x\cong 1$, according to Eq. (10) we conclude that the critical temperature depends only on *χ* and is the same as for *x*_0_ = 0.

Let us now verify the characteristic behavior of the critical temperatures under increasing pressure shown in Fig. 1. Experimental data for high-*T*_c_ superconductors such as LBCO^58^, mercury-based high-*T*_c_ compounds^59^, as well as H_3_S^17,60^ reveal behaviour similar to type-(a). A suitable choice of the values for the parameters *x*_0_, *T*_c_(0) and *T*_c_(1) can result in a quite good quantitative agreement. Also, the experimental data for sulfur hydride (H_2_S) or sulfur deuteride (D_2_S) with the superconducting critical temperature above 200 K (under high hydrostatic pressure)^17,61^ reveals that at in the high-*T*_c_ range, the critical temperature vs. pressure dependence is compatible with the type (a).

Furthermore. the experimental data for the pressure shift of the critical temperature for a single crystal of rhenium (which are similar to the thallium data), as well as the pressure shift of the critical temperature for Re and Re-Os alloys at very low concentrations (less than 0.11%)^62^, reveal the relationship corresponding to the case (b).

On the other hand, the critical temperature for V_3_Si^63^, Nb_3_Sn^64^, MgB_2_^65^, SrAlSi^66^, and Re-Os alloys (with concentrations greater than 2.75%)^62^ decreases linearly with pressure. Therefore, this behavior corresponds to the type (c) when ${x}_{0}\gg \kappa p$.

Finally, the pressure dependence of the critical temperature for V_0.54_Ru_0.4_6^67^ and CaAlSi^66^ coincides with that of the type-(d), with the pressure-induced change of the critical temperature in V_3_Si^63^ corresponding to the linear part of this case.

Note that a similar picture of the *T*_c_ vs *p*, that is of type-(d), was obtained for H_3_S_0.925_P_0.075_ in the range of pressure from 150 to 250 GPa and critical temperatures *c*.*a*. between 225 and 250 K^68^.

Moreover, ref.^66^ quotes the values of the pressure coefficient for SrAlSi (*c*_*p*_ = −0.12 K/GPa) and the pressure coefficient at zero pressure for CaAlSi (*c*_*p*_ = 0.21 K/GPa).

In order to analyze the increase of the critical temperature under high pressure^69–74^, one should assume that the peak in the density of states is located below the Fermi level, *i*.*e*. *x*_0_ < 0 and |*x*_0_| ~ 0.1 ÷ 1, which corresponds to the case (a) shown in Fig. 1. Then the critical temperature significantly increases for sufficiently high pressure until *p* ≤ −*x*_0_/*κ*. If the pressure exceeds the value of −*x*_0_/*κ*, the critical temperature decreases and it can become very small in the higher pressure range.

In order to illustrate the versatility of the developed approach, in Table 1 we present five examples of values for a set of parameters characterizing type-(a) superconductors, and one example corresponding to type (b). These parameters are: the critical temperature at zero pressure – *T*_c_(0), the supreme critical temperature achieved at pressure *p*_*m*_ – *T*_c_(1), the value of the pressure *p*_*m*_ itself, and the pressure coefficient (at zero pressure) *c*_*p*_(*x*_0_). The corresponding values of the model parameters *x*_0_, *χ*, and *κ* are derived from Eqs (12) and (15), and the relation *κ* = −*x*_0_/*p*, respectively.

**Table 1:** Example values of model parameters: *x*_0_, *χ*, and *κ*, determined based on the experimentally measurable quantities: *T*_c_(0), *T*_c_(1), *p*_*m*_, and *c*_*p*_(*x*_0_).

Example	*T*_c_(0)	*T*_c_(1)	*p*_*m*_	*c*_*p*_(*x*_0_)	*x*_0_	*χ*	*κ*	Representative superconductor
	[K]	[K]	[GPa]	[K/GPa]	[1]	[1]	[GPa^−1^]	
(1)	12	37	7	1.02	−4.0	1.501	0.571	*α*–FeSe
(2)	7	30	6.5	1.33	−3.6	2.014	0.554	FeSe
(3)	13.5	26.2	2	9.752	−1.8	1.399	0.90	FeSe_0.5_Te_0.5_
(4)	32	40	1.33	4.006	−2.4	0.378	1.805	La_2−*x*_Ba_*x*_CuO_4−*y*_
(5)	137	164	34	1.79	−0.653	1.481	0.019	Hg-1223
(6)	1.695	1.684	0.65	−0.03	−0.55	−0.072	0.846	Re

The sets of values correspond to type-(a) behavior for (1)–(5), and type-(b) behavior for (6). They are matched with representative superconducting materials listed in the last column of the table.

**Figure 3:**
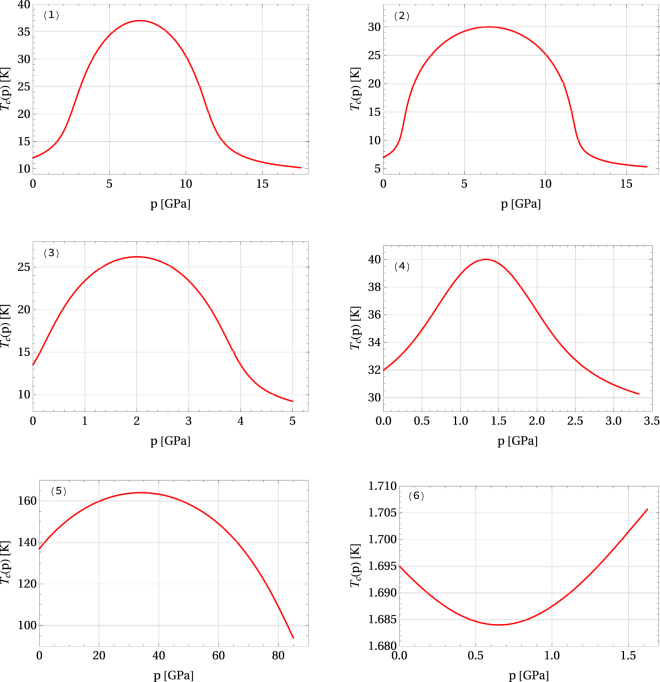
Examples of the critical temperature *T*_c_(*p*) vs. pressure *p* = *αp*_*m*_ dependence for *α* ∈ [0, 2.5] and a few postulated sets of values of *T*_c_(0), *T*_c_(1), *p*_*m*_, and *c*_*p*_(*x*_0_) given in Table 1. Plots (1)–(5) illustrate type-(**a**) behavior, whereas plot (6) depicts a type-(**b**) curve.

In Fig. 3, based on Eq. (14), we present the dependence of the critical temperature *T*_c_(*α*, *x*_0_) on pressure defined as *αp* for *α* ∈ [0, 2.5], for six cases given in Table 1. Comparing the plots (1)–(6) obtained within our simple model with some other results in literature, one should state that they coincide to a satisfactory degree in a quite wide range of pressure values. Namely, plots (1) and (2) correspond to the curves obtained for the superconductors *α*–FeSe (orthorhombic crystal structure) and high-quality FeSe polycrystal presented in refs^71,74^. The dome-shaped dependence of the critical temperature with the threshold value of the external pressure at 6.5 7 GPa is well reproduced within our simple one-peak model with the values of parameters listed in the first two rows of Table 1. The threshold pressure defines the value at which a widely reported four-fold increase in the pressure occurs. It is worth to notice that above 6 GPa this sudden enhancement of superconductivity is accompanied by a suppression of magnetic order^75^, which may also contribute to fact that our model can still be applied here.

Moreover, plot (3) reproduces the relation for FeSe_05_Te_0.5_^76^, and plot (4) coincides with the curve for the HTC superconductor La_2−*x*_Ba_*x*_CuO_4−*y*_^58^. Finally, plot (5) is in agreement with the corresponding relation for Hg-1223^59^, and plot (6) reproduces the relation for a single crystal of rhenium^62^.

### Zero-temperature case

Considering the case of *T* = 0 for ${\varrho }_{j}(x)=2\chi \delta (x-{x}_{0})$ with the pressure effect included, we need to redefine the symbols introduced in Eq. (5) as follows: *x* = *ξ*/2*T*_c_(*χ*, *x*_0_, *p*), *x*_*p*,*χ*_ = *ξ*_*p*_/2*T*_c_(*χ*, *x*_0_, *p*), and *D*(0, *χ*, *x*_0_, *p*) = Δ(0, *χ*, *x*_0_, *p*)/2*T*_c_(*χ*, *x*_0_, *p*). Then Eq. (5) assumes the form

16 $$\frac{1}{g}=\frac{{\nu }_{0}}{2}\,{\int }_{-{x}_{p,\chi }}^{{x}_{p,\chi }}\,\frac{dx\,[1+2\chi \delta (x-\tau {x}_{0})]}{\sqrt{{(x+\tau \kappa p)}^{2}+{D}^{2}(0,\chi ,{x}_{0},p)}},$$

where we again note that *x*_0_ and *κ* must be fixed with respect to the critical temperature *T*_c_(*χ*, *x*_0_, 0) defined for *p* = 0. Evaluating the integral and omitting terms of order (*Qp*/*ξ*_*p*_)^2^ we obtain

17 $$\frac{1}{g{\nu }_{0}}=\,\mathrm{ln}\,\frac{{\xi }_{p}/{T}_{{\rm{c}}}(\chi ,{x}_{0},p)}{D(0,\chi ,{x}_{0},p)}+\frac{\chi }{\sqrt{{[\tau ({x}_{0}+\kappa p)]}^{2}+{D}^{2}(0,\chi ,{x}_{0},p)}},$$

where the values of *D*(0, *χ*, *x*_0_, *p*) and *T*_c_(*χ*, *x*_0_, *p*) vary along with *x*_0_ and *p*.

Let us now find the free energy difference by means of Eq. (6). After some algebra we get

18 $$\begin{array}{rcl}{\rm{\Delta }}F(0,\chi ,{x}_{0},p) & = & -\frac{{\nu }_{0}}{4}{{\rm{\Delta }}}^{2}(0,\chi ,{x}_{0},p)-2{\nu }_{0}\chi \,{T}_{c}^{2}(\chi ,{x}_{0},p)\\  &  & \times [2\sqrt{{[\tau ({x}_{0}+\kappa p)]}^{2}+{D}^{2}}-2\tau |{x}_{0}+\kappa p|-\tfrac{{D}^{2}}{\sqrt{{[\tau ({x}_{0}+\kappa p)]}^{2}+{D}^{2}}}],\end{array}$$

and here

$$D=\frac{{\rm{\Delta }}(0,\chi ,{x}_{0},p)}{2{T}_{{\rm{c}}}(\chi ,{x}_{0},p)}.$$

Assuming that the same parameters *ν*_0_, *g* and *ξ*_*p*_ are fixed for both the BCS–like model (*χ* = 0) and the model under discussion (*χ* ≠ 0, *p* > 0), Eq. (17) one yields

19 $${\rm{\Delta }}(0,\chi ,{x}_{0},p)={\rm{\Delta }}(0,0,0)\,\exp \,\{\frac{\chi }{\sqrt{{[\tau ({x}_{0}+\kappa p)]}^{2}+{[\frac{{\rm{\Delta }}(0,\chi ,{x}_{0},p)}{2{T}_{{\rm{c}}}(\chi ,{x}_{0},p)}]}^{2}}}\}.$$

## The Ratio ${{\boldsymbol{ {\mathcal R} }}}_{{\bf{1}}}$(*χ*)

The derived Eqs (10) and (19) allow us to discuss the ratio ${ {\mathcal R} }_{1}$ as a function *χ*, *x*_0_, and *p*. Taking into account that ${ {\mathcal R} }_{1}(\chi ,{x}_{0},p)=2{\rm{\Delta }}(0,\chi ,{x}_{0},p)/{T}_{{\rm{c}}}(\chi ,{x}_{0},p)$, we have the following equation

20 $${ {\mathcal R} }_{1}(\chi ,{x}_{0},p)={ {\mathcal R} }_{1}\,\exp \,\{\chi [\tfrac{4}{\sqrt{{[4\tau ({x}_{0}+\kappa p)]}^{2}+{ {\mathcal R} }_{1}^{2}(\chi ,{x}_{0},p)}}-\tfrac{\tanh \,[\tau ({x}_{0}+\kappa p)]}{\tau ({x}_{0}+\kappa p)}]\}\,,$$

where *τ* must be found from Eq. (11) first. We find ${ {\mathcal R} }_{1}(\chi ,{x}_{0},p)$ numerically, assuming that $3\leqslant { {\mathcal R} }_{1}(\chi ,{x}_{0},p)\leqslant 4$ and including that for the BCS case (*χ* = 0) ${ {\mathcal R} }_{1}=3.528$. Comparing the obtained results with experimental data for some superconducting superconductors^16,21–25,77–79^ we can find the corresponding value of the parameter *χ*, and hence calculate the values of ${ {\mathcal R} }_{1}(\chi )$ for these systems.

**Figure 4:**
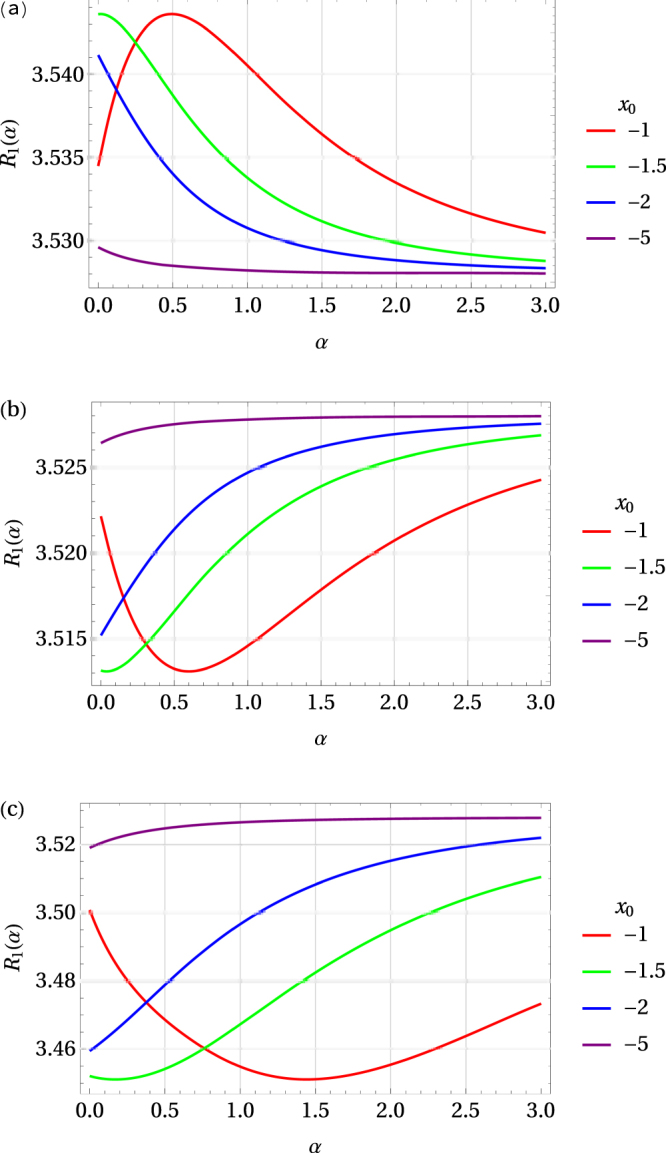
Ratio ${ {\mathcal R} }_{1}$ as a function of pressure for superconducting systems with *χ* = −0.15 (**a**), 0.15 (**b**), and 0.85 (**c**) and different values of *x*_0_ < 0 indicated in the legends. *α* = *κp*/|*x*_0_| is a dimensionless positive scaling parameter for pressure.

**Figure 5:**
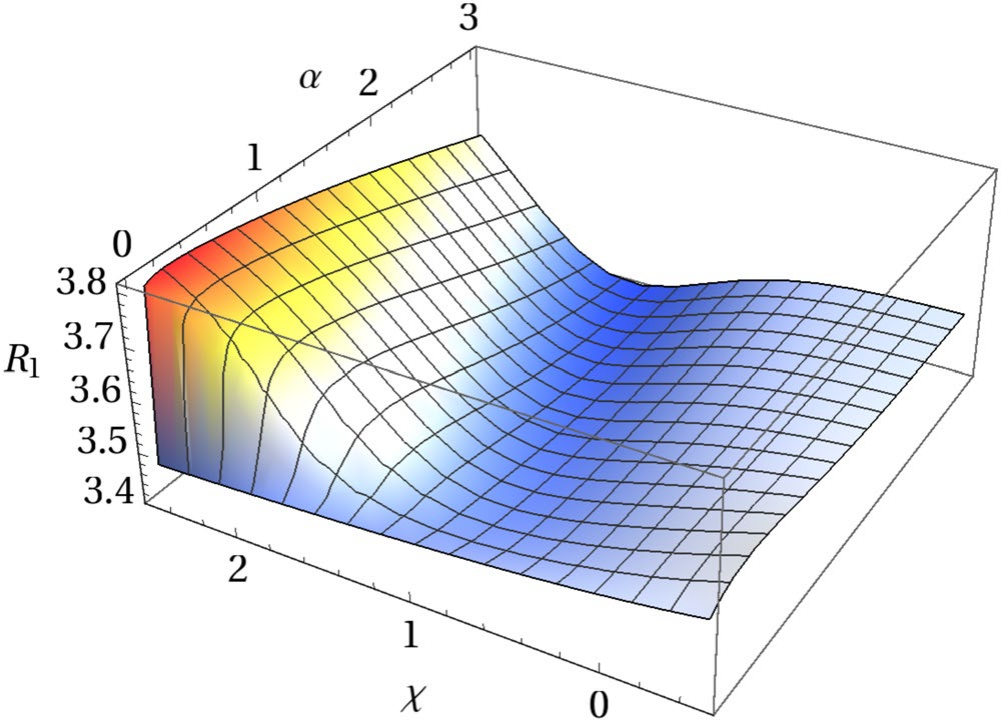
Ratio ${ {\mathcal R} }_{1}$ as a function of pressure and the parameter *χ* for *x*_0_ = −1. Here −0.5 ≤ *χ* ≤ 2.5 and 0 ≤ *α* ≤ 3, with *x*_0_ + *κp* = *x*_0_(1 − *α*).

The ratio ${ {\mathcal R} }_{1}$ as a function of pressure for superconducting systems with *χ* = −0.15, 0.15, and 0.85 is shown in Fig. 4. With the peak in the density of states moving farther away from the Fermi level (*i*.*e*. with increasing |*x*_0_|), the maximum (or minimum, for *χ* > 0) in ${ {\mathcal R} }_{1}$ shifts towards the region of lower pressure values. Moreover, with the increasing value of the parameter *χ* that maximum (minimum) flattens out. This tendency is also confirmed in Fig. 5 showing the 3D graph of the ratio ${ {\mathcal R} }_{1}$ as a function of pressure and the parameter *χ* for a system with *x*_0_ = −1.

## The Ratio ${{\boldsymbol{ {\mathcal R} }}}_{{\bf{2}}}$(*χ*)

The ratio ${ {\mathcal R} }_{2}\equiv {\rm{\Delta }}C({T}_{{\rm{c}}})/{C}_{{\rm{N}}}({T}_{{\rm{c}}})$, where Δ*C*(*T*_c_) = *C*_S_(*T*_c_) − *C*_N_(*T*_c_) defines the jump of the heat capacity between the superconducting and the normal phase at the transition temperature as a function of *χ*, *x*_0_ and *p*. In order to find its value, we need to consider Eq. (9) and use Eq. (6) in the sub-critical temperature range, when ${\varrho }_{j}(x)$ = 2*χδ*(*x* − *x*_0_).

### Sub-critical temperature range

In the sub-critical temperature range, *i*.*e*. for $T\lesssim {T}_{{\rm{c}}}(\chi )$, with the pressure included, we have to refer the discussion to the case *p* = 0, where *x*_0_ and *κ* are steady. In this region, the magnitude of Δ(*T*, *χ*, *x*_0_, *p*)/2*T* is small and, in the first order of the perturbation method, Eq. (9) can be transformed to the form

21 $$\begin{array}{rcl}\frac{1}{g{\nu }_{0}} & = & \mathrm{ln}\,\frac{{\xi }_{p}}{2T}+\,\mathrm{ln}\,\frac{4{e}^{C}}{\pi }-a\,{[\frac{{\rm{\Delta }}(T,\chi ,{x}_{0},p)}{2{T}_{{\rm{c}}}(\chi ,{x}_{0},p)}]}^{2}\\  &  & +\chi \,\frac{\tanh \,[\tau ({x}_{0}+\kappa p)]}{{x}_{0}+\kappa p}-\frac{1}{3}\chi \varphi \,(\tau ({x}_{0}+\kappa p))\,\,{[\frac{{\rm{\Delta }}(T,\chi ,{x}_{0},p)}{2{T}_{c}(\chi ,{x}_{0},p)}]}^{2},\end{array}$$

where

$$a=\frac{7\zeta (3)}{2{\pi }^{2}}=0.426,\,\phi (x)=\frac{3}{2{x}^{3}}\,(\tanh \,x-x\,{\cosh }^{-2}\,x),$$

and *φ*(*x*) ≤ 1 is an even and positive function of the real variable *x*. Moreover, *φ*(0) = 1 and it quickly approaches 0 as |*x*| increases from 0 to ∞. Taking into account the form of Eq. (21) obtained for *T* = *T*_c_(*χ*, *x*_0_, *p*) (Δ = 0), and plugging it into Eq. (21), we find

22 $$\begin{array}{rcl}{\rm{\Delta }}(T,\chi ,{x}_{0},p)) & = & \frac{2{T}_{{\rm{c}}}(\chi ,{x}_{0},p)}{\sqrt{a[1+\frac{\chi }{3a}\,\phi \,(\tau ({x}_{0}+\kappa p))]}}\sqrt{1-\frac{T}{{T}_{{\rm{c}}}(\chi ,{x}_{0},p)}}\\  & = & \frac{3.063\,{T}_{{\rm{c}}}(\chi ,{x}_{0},p)}{\sqrt{1+0.782\,\chi \,\phi \,(\tau ({x}_{0}+\kappa p))}}\sqrt{1-\frac{T}{{T}_{{\rm{c}}}(\chi ,{x}_{0},p)}}.\end{array}$$

Let us complete the discussion in this section with the equation for the free energy difference Δ*F* derived according to Eq. (6) in the first order of the perturbation method. Using Eqs (21) and (22), after some algebra we obtain

23 $$\begin{array}{rcl}{\rm{\Delta }}F(T,\chi ,{x}_{0},p) & = & -\frac{{\nu }_{0}{T}_{{\rm{c}}}^{2}(\chi ,{x}_{0},p)}{a[1+\frac{\chi }{3a}\,\phi \,(\tau ({x}_{0}+\kappa p))]}\,{[1-\frac{T}{{T}_{{\rm{c}}}(\chi ,{x}_{0},p)}]}^{2}\\  & = & -\frac{2.346\,{\nu }_{0}{T}_{{\rm{c}}}^{2}(\chi ,{x}_{0},p)}{1+0.782\,\chi \,\phi (\tau ({x}_{0}+\kappa p))}\,{[1-\frac{T}{{T}_{{\rm{c}}}(\chi ,{x}_{0},p)}]}^{2}.\end{array}$$

### Specific heat jump

Using standard thermodynamic relations for the free energy difference (23), we can find the normalized heat capacity difference Δ*C*(*T*)/*C*_N_(*T*_c_) which, in the first-order perturbation method, determines the normalized heat capacity jump at *T* = *T*_c_(*χ*, *x*_0_ + *κp*). Taking into account that the characteristic ratio ${ {\mathcal R} }_{2}\equiv {\rm{\Delta }}C({T}_{{\rm{c}}})/{C}_{{\rm{N}}}({T}_{{\rm{c}}})$, in the case under discussion we have

24 $${ {\mathcal R} }_{2}(\chi ,{x}_{0},p)=\tfrac{6\,\exp \,[\chi \,\tfrac{\tanh (\tau ({x}_{0}+\kappa p))}{\tau ({x}_{0}+\kappa p)}]}{{\pi }^{2}a[1+\tfrac{\chi }{3a}\,\phi \,(\tau ({x}_{0}+\kappa p))]}=\tfrac{1.426\,\exp \,[\chi \,\tfrac{\tanh (\tau ({x}_{0}+\kappa p))}{\tau ({x}_{0}+\kappa p)}]}{1+0.782\chi \,\phi (\tau ({x}_{0}+\kappa p))}.$$

where ${C}_{{\rm{N}}}({T}_{{\rm{c}}})=\frac{1}{3}{\nu }_{0}{\pi }^{2}{T}_{{\rm{c}}}$ must be constant and independent of *χ*. Therefore, *T*_c_ ≡ *T*_c_(0, 0, 0) and in order to eliminate *T*_c_(*χ*, *x*_0_, *p*)/*T*_c_(0, 0, 0) from Eq. (24) we have used Eq. (10). The magnitude of this jump for *p* = 0 and *x*_0_ = 0 at the critical temperature decreases from 1.459 to 1.380 for −0.576 < *χ* < −0.279 and it starts to increase for *χ* > −0.279. If *χ* = 0, it is equal to the BCS value of 1.426, and for *χ* > 0 the magnitude of the jump is still increasing.

According to the experimental data for the pressure dependence of the critical temperature given in ref.^16^, the superconductors SrAlSi and MgB_2_ have been identified as type-(c) with *x*_0_ > 0 and *χ* > 0, and CaAlSi — as type-(d) with *x*_0_ > 0 and *χ* < 0. However, the experimental data for the normalized heat capacity of CaAlSi, SrAlSi and MgB_2_ at the normal pressure (*p* ≈ 0), suggest that the superconductors SrAlSi and MgB_2_ should be of type-(b) with *x*_0_ < 0 and *χ* < 0 whereas CaAlSi — of type-(a) with *x*_0_ < 0 and *χ* > 0. Then the normalized heat capacity jump exceeds the BCS value for *χ* > 0 and its magnitude remains smaller than the BCS value for *χ* < 0. However, if these superconductors were of type-(a) or -(b), there would need to exist some values of the external pressure for which the critical temperatures achieve their maximum or minimum, respectively.

### Additional relations and comments

Based on Eq. (20), we can derive *χ* in dependence on ${ {\mathcal R} }_{1}(\chi ,{x}_{0},p)$. Plugging it into Eq. (24) we find ${ {\mathcal R} }_{2}$ as a function ${ {\mathcal R} }_{1}$ in the form

25 $$\begin{array}{rcl}{ {\mathcal R} }_{2} & = & 1.426\,\exp \,[\tfrac{\tanh (\tau ({x}_{0}+\kappa p))}{\tau ({x}_{0}+\kappa p)}\tfrac{\sqrt{{[4\tau ({x}_{0}+\kappa p)]}^{2}+{ {\mathcal R} }_{1}^{2}}\,\mathrm{ln}(0.283\,{ {\mathcal R} }_{1})}{4-\tfrac{\tanh (\tau ({x}_{0}+\kappa p))}{\tau ({x}_{0}+\kappa p)}\,\sqrt{{[4\tau ({x}_{0}+\kappa p)]}^{2}+{ {\mathcal R} }_{1}^{2}}}]\\  &  & \times \,{[1+\tfrac{0.782\phi (\tau ({x}_{0}+\kappa p))\sqrt{{\mathrm{[4}\tau ({x}_{0}+\kappa p)]}^{2}+{ {\mathcal R} }_{1}^{2}}\mathrm{ln}\mathrm{(0.283}{ {\mathcal R} }_{1})}{4-\tfrac{\tanh (\tau ({x}_{0}+\kappa p))}{\tau ({x}_{0}+\kappa p)}\sqrt{{\mathrm{[4}\tau ({x}_{0}+\kappa p)]}^{2}+{ {\mathcal R} }_{1}^{2}}}]}^{-1}.\end{array}$$

Note that the ratio ${ {\mathcal R} }_{2}$ vs ${ {\mathcal R} }_{1}$ obtained for *x*_0_ = 0 and *p* = 0 in ref.^39^ coincides with that of type-(a) and that of type-(b) derived for the maximum or the minimum critical temperature *T*_c_(1), respectively, *i*.*e*. achieved when *x*_0_ + *κp* = 0. Then Eq. (25) simplifies to

26 $${ {\mathcal R} }_{2}=\frac{1.426\,(4-{ {\mathcal R} }_{1})}{4-{ {\mathcal R} }_{1}+0.782\,{ {\mathcal R} }_{1}\,\mathrm{ln}(0.283\,{ {\mathcal R} }_{1})}\,\exp \,[\frac{{ {\mathcal R} }_{1}\,\mathrm{ln}(0.283\,{ {\mathcal R} }_{1})}{4-{ {\mathcal R} }_{1}}].$$

It is worth to emphasize that this relation has been verified by comparing with experimental data for some low-temperature superconducting materials^39^. On the other hand, for some superconductors that can be of type-(a), -(b), -(c), or -(d), in order to derive the ratio ${ {\mathcal R} }_{2}$ vs ${ {\mathcal R} }_{1}$ for a particular superconductor and for a given value of *p*, the set of three values of *x*_0_, *κ*, and *τ* must be known. However, for all such sets for which the expression *τ*(*x*_0_ + *κp*) has exactly the same value, the ratio ${ {\mathcal R} }_{2}$ vs ${ {\mathcal R} }_{1}$ can be easily found from Eq. (25). Moreover, since the rhs of Eq. (25) is an even function of *τ*(*x*_0_ + *κp*), the results obtained for ±*τ*(*x*_0_ + *κp*) are identical.

**Figure 6:**
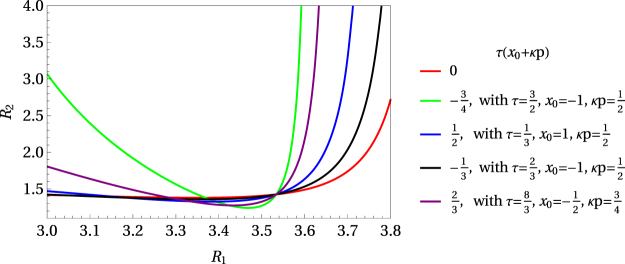
Ratio ${ {\mathcal R} }_{2}$ vs ${ {\mathcal R} }_{1}$ for the values of *τ*(*x*_0_ + *κp*) equal to $0,-\,\frac{3}{4},\frac{1}{2},-\,\frac{1}{3},\frac{2}{3}$.

**Figure 7:**
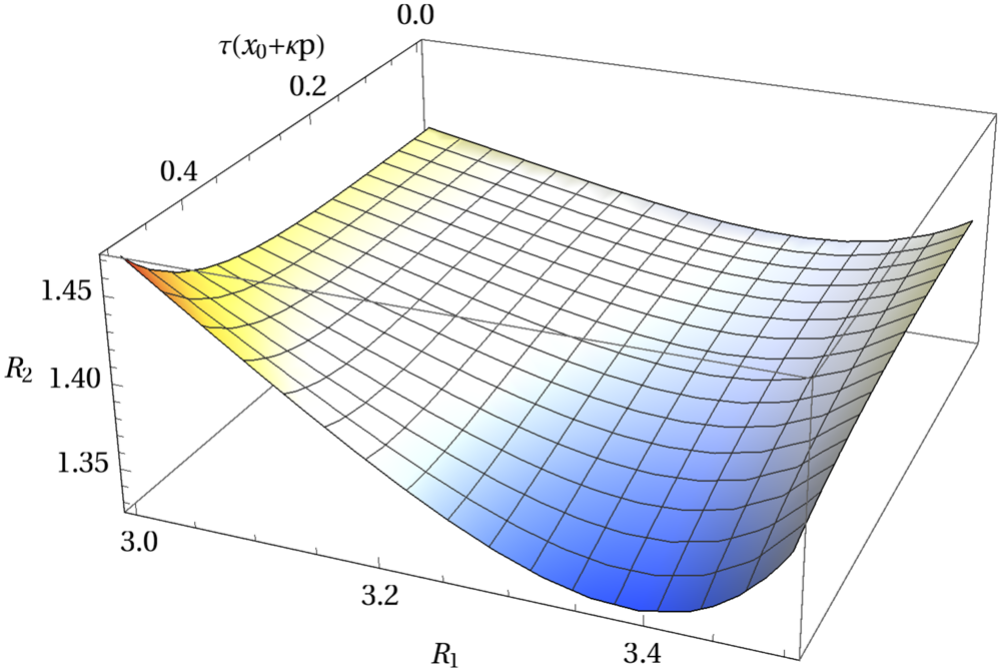
Ratio ${ {\mathcal R} }_{2}$ vs ${ {\mathcal R} }_{1}$ for 0 ≤ *τ*(*x*_0_ + *κp*) ≤ 0.5.

In Fig. 6 the graphs of the ratio ${ {\mathcal R} }_{2}$ vs ${ {\mathcal R} }_{1}$ are presented for the case *x*_0_ + *κp* = 0 and for given hypothetical values of *τ*, *x*_0_, and *κp*. Some points on these graphs will refer to different superconductors if the values of the expression *τ*(*x*_0_ + *κp*) for the corresponding parameters *τ*, *x*_0_, and *κp* coincide. Additionally, in Fig. 7, the ratio ${ {\mathcal R} }_{2}$ vs ${ {\mathcal R} }_{1}$ for 0 ≤ *τ*(*x*_0_ + *κp*) ≤ 0.5 is plotted. Please note that, if *p* = 0 then *τ* = 1, and such a case corresponds to a narrow fluctuation shifted a distance *x*_0_ from the Fermi level. It is an extension of the approach discussed in ref.^39^.

Using the numerical form of ${ {\mathcal R} }_{1}(\chi ,{x}_{0},p)$ derived from Eq. (20) we can rewrite Eq. (19) in the form

27 $${\rm{\Delta }}(0,\chi ,{x}_{0},p)={\rm{\Delta }}(0,0,0)\,\exp \,[\frac{4\chi }{\sqrt{{[4\tau ({x}_{0}+\kappa p)]}^{2}+{ {\mathcal R} }_{1}^{2}(\chi ,{x}_{0},p)}}],$$

and hence find the free energy difference (12) as

28 $$\begin{array}{rcl}{\rm{\Delta }}\,F(0,\chi ,{x}_{0},p) & = & -\frac{{\nu }_{0}}{4}\,{{\rm{\Delta }}}^{2}(0,0,0)\,\exp \,\{\tfrac{8\chi }{{[4\tau ({x}_{0}+\kappa p)]}^{2}+{ {\mathcal R} }_{1}^{2}(\chi ,{x}_{0},p)}\}\\  &  & \times \,\{1+\tfrac{8\chi }{{ {\mathcal R} }_{1}^{2}(\chi ,{x}_{0},p)}[2\sqrt{{[4\tau ({x}_{0}+\kappa p)]}^{2}+{ {\mathcal R} }_{1}^{2}(\chi ,{x}_{0},p)}\\  &  & -\,8\tau |{x}_{0}+\kappa p|-\tfrac{{ {\mathcal R} }_{1}^{2}(\chi ,{x}_{0},p)}{\sqrt{{[4\tau ({x}_{0}+\kappa p)]}^{2}+{ {\mathcal R} }_{1}^{2}(\chi ,{x}_{0},p)}}]\}.\end{array}$$

**Figure 8:**
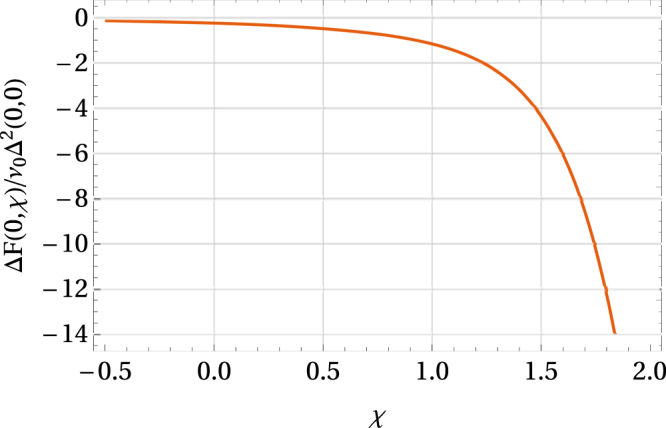
Free energy difference as a function of *χ*. Here *x*_0_ + *κp* = *x*_0_(1 − *α*) with *x*_0_ = −1 and *α* = 0.5.

Eqs (27) and (28) allow us to evaluate Δ(0, *χ*, *x*_0_, *p*) and Δ*F*(0, *χ*, *x*_0_, *p*) numerically as functions of *χ*, cf. Fig. 8.

Moreover, based on Eqs (10), (22) and (23) we can also estimate the changes of Δ(*T*, *χ*, *x*_0_, *p*) in dependence on *χ*, *x*_0_, and *p* for sub-critical temperatures ($T\lesssim {T}_{{\rm{c}}}(\chi ,{x}_{0},p)$). Namely, in the first-order perturbation method,

29 $${\rm{\Delta }}(T,\chi ,{x}_{0},p)={\rm{\Delta }}(0,0,0)\,{\rm{\Phi }}(\chi ,{x}_{0}+\kappa p)\sqrt{1-\frac{T}{{T}_{{\rm{c}}}(\chi ,{x}_{0},p)}},$$

where

$${\rm{\Phi }}(\chi ,{x}_{0}+\kappa p)=\tfrac{2\,{e}^{C}\,\exp \,[\chi \tfrac{\tanh (\tau ({x}_{0}+\kappa p))}{\tau ({x}_{0}+\kappa p)}]}{\pi \sqrt{a[1+\tfrac{\chi }{3a}\,\phi (\tau ({x}_{0}+\kappa p))]}}=1.737\tfrac{\exp \,[\chi \tfrac{\tanh (\tau ({x}_{0}+\kappa p))}{\tau ({x}_{0}+\kappa p)}]}{\sqrt{1+0.782\,\chi \,\phi (\tau ({x}_{0}+\kappa p))}},$$

and hence the free energy difference

30 $$\begin{array}{rcl}{\rm{\Delta }}\,F(T,\chi ,{x}_{0},p) & = & -\frac{{\nu }_{0}}{4}\,{{\rm{\Delta }}}^{2}(0,0,0)\,{{\rm{\Phi }}}^{2}(\chi ,{x}_{0}+\kappa p){[1-\frac{T}{{T}_{{\rm{c}}}(\chi ,{x}_{0},p)}]}^{2}\\  & = & -\frac{{\nu }_{0}}{4}\,{{\rm{\Delta }}}^{2}(T,\chi ,{x}_{0},p)[1-\frac{T}{{T}_{{\rm{c}}}(\chi ,{x}_{0},p)}].\end{array}$$

**Figure 9:**
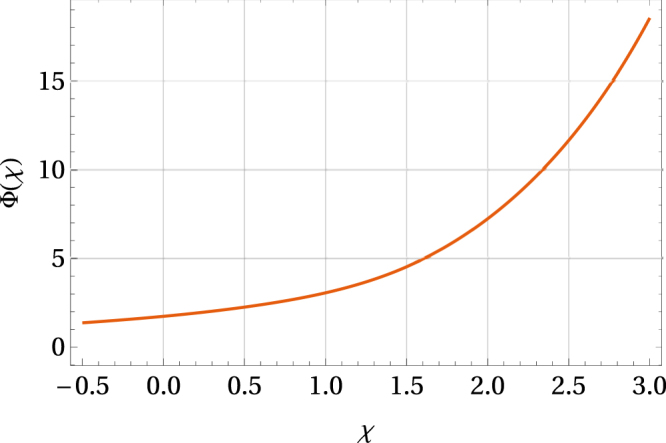
The dependence of the function Φ on the parameter *χ*. Here *x*_0_ + *κp* = *x*_0_(1 − *α*) with *x*_0_ = −1 and *α* = 0.5.

The form of Φ(*χ*, *x*_0_ + *κp*) is given in Fig. 9.

Note that in the superconducting state Δ*F*(*T*, *χ*, *x*_0_, *p*) given by Eq. (23) must be negative, hence we demand the parameter *χ* > −1.279/*φ*(*τ*(*x*_0_ + *κp*)), remembering that *φ*(*x*) ≤ 1.

## Conclusions

In the paper, we have identified four universal types of the response of superconductors to an external high pressure, in terms of the dependence of the critical temperature on pressure. We have found and discussed the forms of the superconducting gap, free energy difference, and specific heat difference for *T* = 0 and in the sub-critical temperature range $T\lesssim {T}_{{\rm{c}}}$, with the external high pressure included. A range of numerical results shows that experimental data can be used to find the critical temperature as a function of pressure and discuss other properties of superconducting systems under high pressure.

Similar thermodynamic relations were also obtained in refs^80,81^, where the pressure was included in terms of lattice deformation. According to the results presented there, high pressure modifies peaks in the density of states to a small (negligible) degree.

The presented simplified model is based on the assumption that the crystal structure of a superconductor is stable under high pressure. In general, however, in some materials high pressure can induce structural phase transitions, and properties of such systems may change drastically. Then, the one-particle dispersion relation *ξ*_**k**_ assumes a new, stable form, and if the superconductivity is not suppressed, the new system can be analyzed again within the conformal transformation method, using the approach presented in this paper. However, compared to the system before the structural phase transition has taken place, it may reveal quite different properties. In particular, one type of dependence of the critical temperature on pressure may be replaced by another when the pressure is being increased. A similar effect on the critical temperature of the high-*T*_c_ superconducting system YBCO should be observed when the oxygen content is being adjusted^82,83^.

The agreement of the results obtained within the simple model with experimental data and ab-initio calculations, suggests that effects included in the model dominate when the critical temperature changes with increasing external pressure. We may then conclude that some other effects, that may have been included in calculations, give almost negligible contribution. We emphasize that, within the used formalism, all other extra effects find their expression in the complex form of the scalar field of the density of states. This scalar field, for *s*-wave superconducting systems, simply reduces to the usual density of states, enriched by the presence of strong fluctuations. In a simple model, these fluctuations are replaced by a single narrow peak. This approach allows us to identify conditions that must be satisfied for the critical temperature to change with pressure, as well as the form of this change. Therefore the simple model should prove to be useful in the quest for superconductors with increasingly higher critical temperatures.

The success of the simple model presented in our paper originates from the following fact: The conformal transformation method allows us to transform any model (formalism) describing a superconductor, including a HTSC, with a given symmetry defined in the reciprocal space (or transformed to that space) to a mathematically fully equivalent model in an isotropic reciprocal space. At this stage, mathematical transformations are reversible. Although the space becomes isotropic, that does not mean that the description is simplified, because the usual electronic density of states becomes an involved function of 2 (or 3) variables a scalar field of the density of states that now carries all information about the system.

In the approach presented in this paper it was enough to take into account a model form of ${\varrho }_{j}(x)$, where a single narrow fluctuation (a peak) located a distance *x*_0_ from the Fermi level in the density of states appears, represented by 2*χδ*(*x* − *x*_0_).

The position of this peak *x*_0_ and its weight *χ*, have allowed us to identify four universal types of the response of superconductors, in terms of the dependence of the critical temperature on increasing external pressure, also confirmed by experimental data. We would also like to emphasize that the forms of the density of states vs. energy, obtained by ab-initio calculations presented in refs^16,68,84–87^, include strong fluctuations, with the main ones placed below the Fermi level, in agreement with our simple model.

Although here the parameters *χ* and *x*_0_ are independent of the pressure *p*, in more detailed studies, one might also adjust their values for high pressure. Also, in reference to the quoted ab-initio results, one might introduce a number of various fluctuations (peaks) in the density of states in the form ${\sum }_{i}\,2\,{\chi }_{i}\,\delta (x-{x}_{i})\,$, where *x*_*i*_ is the distance of the *i*-th fluctuation from the Fermi level and *x*_*i*_ as well as *χ*_*i*_ can be either positive or negative^80^. Such an approach should make possible to improve the accuracy of the description of thermodynamic properties of some simple and composed superconducting systems^7,21–23,26^. However, the most promising case seems to be the one when just two fluctuations are included.

As a closing remark, let us emphasize that although the approach presented in this paper corresponds to the Van Hove Scenario, it is obtained in a quite general manner by applying the more general conformal transformation method, that includes the Van Hove Scenario as its special case^3,7,8,20,27,35,41,88^.

### Data availability

All data generated or analyzed during this study are included in this published article.

## Electronic supplementary material

Appendix
