# Supplementary material for: Simple analytical model of the effect of high pressure on the critical temperature and other thermodynamic properties of superconductors
Source: Sci Rep. 2018 May 16;8:7709. doi: 10.1038/s41598-018-26029-9 (PMC5955910; doi:10.1038/s41598-018-26029-9)
Supplement: Supplementary file 1 — Appendix [file 41598_2018_26029_MOESM1_ESM.pdf]

Simple analytical model of the effect of high pressure on the critical temperature and other thermodynamic properties of superconductors

MATEUSZ KRZYZOSIAK,[1] RYSZARD GONCZAREK,[2]
ADAM GONCZAREK,[3] and LUCJAN JACAK[2]

[1] *University of Michigan–Shanghai Jiao Tong University Joint Institute,*
800 Dongchuan Rd, Shanghai 200240, China
m.krzyzosiak@sjtu.edu.cn

[2] *Faculty of Fundamental Problems of Technology, Wrocław University of Technology,*
Wybrzeże Wyspiańskiego 27, 50-370 Wrocław, Poland

[3] *Faculty of Computer Science and Management, Wrocław University of Technology,*
Wybrzeże Wyspiańskiego 27, 50-370 Wrocław, Poland

APPENDIX

TABLE I: List of symbols and their definitions.

Symbol	Definition – general case
x_0	position of the fluctuation in DoS (relative to the Fermi level)
χ	height of the fluctuation
p	external (hydrostatic) pressure
$\xi_{\mathbf{k}}(0)$	dispersion relation for pressure-free system
v	(real-space) specific volume occupied by a charge carrier
Q	$= \left[\frac{\partial}{\partial p}\xi_{\mathbf{k}}(p)\right]_{\mathbf{k}=\mathbf{k}_\mathrm{F},\, p=0}$
κ	$= Q/2T_\mathrm{c}(\chi, x_0, 0)$
$T_\mathrm{c}(\chi, x_0, 0)$	critical temperature for pressure-free system
$T_\mathrm{c}(\chi, x_0, p)$	critical temperature for system under pressure
τ	$\equiv \tau(\chi, x_0, p) = \frac{T_\mathrm{c}(\chi,x_0,0)}{T_\mathrm{c}(\chi,x_0,p)}$
c_p	$= \left.\frac{d\,T_\mathrm{c}(\chi,x_0,p)}{d\,p}\right\rvert_{p=0}$ pressure coefficient (at zero-pressure)
α	$= \frac{\kappa p}{\lvert x_0\rvert}$, dimensionless positive scaling parameter of pressure
**Symbol**	**Definition – type-(a) and type-(b) (now $x_0 < 0$)**
p_m	$= -\frac{x_0}{\kappa}$, external pressure for which maximum or minimum value of $T_\mathrm{c}(1)$ is achieved
$T_\mathrm{c}(0)$	$= T_\mathrm{c}(\chi, x_0, 0)$, critical temperature under normal conditions ($p = 0$)
$T_\mathrm{c}(1)$	$= T_\mathrm{c}(\chi, x_0, p_m)$, minimum (or maximum) critical temperature under pressure p_m
$T_\mathrm{c}(p)$	critical temperature under pressure p
α	$= \frac{p}{p_m}$, dimensionless positive scaling parameter of pressure
